# USG guided central venous cannulation in ICU : a comparision with conventional approach

**DOI:** 10.1186/2036-7902-7-S1-A1

**Published:** 2015-03-09

**Authors:** A Agarwal, DK Singh, M Tripathi

**Affiliations:** 1 Department of Anesthesiology & Critical Care, All India Institute of Medical Sciences Rishikesh, India; 2 Department of Anesthesiology & Critical Care, Banaras Hindu University, Varanasi, India

## Background

The introduction of portable ultrasonography machines into clinical practice has found widespread usage in the ICUs. They are handy and serve as point of care resource.

Among the common usage in ICU include

- Central venous cannulation

- Assessment of the cause of abdominal distension

- DVT assessment

- Identification of pericardial tamponade

- Valvular pathology & LV function

This saves time and is also safer as it avoids shifting of critically ill patients to other departments.

## Objectives

We performed a study to compare USG & conventional techniques of CVC.

## Material and methods

We performed the study in 60 patients over a period of 3 months. A portable ultrasound machine (Sonosite Micromax) was used with a probe of 7.5 MHz frequency. Cannulation was done using real time imaging. In the conventional method cannulation was done using the landmark approach. The parameters studied included

- Time from completion of draping to successful insertion of needle.

- Number of attempts required.

- Incidence of complications.

## Results

We found that USG approach took lesser time, required lesser attempts and had lower incidence of complications.

## Conclusion

USG guided CVC is easier, quicker & safer than landmark approach.

